# Candidemia Epidemiology and Antifungal Resistance in Critically-ill Patients During and After COVID-19 Pandemic: Comparison Between Two Hospitals in Greece and Türkiye

**DOI:** 10.1007/s11046-026-01086-1

**Published:** 2026-06-16

**Authors:** Esma Eryılmaz Eren, Elias Iosifidis, Hafize Sav, Ilhami Celik, Eleni Mouloudi, Athina Pyrpasopoulou, Charalampos Zarras, Emmanuel Roilides

**Affiliations:** 1 Department of Infectious Diseases and Clinical Microbiology, University of Health Sciences, Kayseri City Training and Research Hospital, 38080 Kayseri, Türkiye; 2 3Rd Department of Pediatrics and Infectious Diseases Unit, Aristotle University of Thessaloniki, Hippokration General Hospital, 54642 Thessaloniki, Greece; 3 Department of Mycology, Kayseri City Training and Research Hospital, 38080 Kayseri, Türkiye; 4 Intensive Care Unit, Hippokration General Hospital, 54642 Thessaloniki, Greece; 5 2nd Department of Propedeutic Medicine and Infectious Diseases Unit, Aristotle University of Thessaloniki, Hippokration General Hospital, 54642 Thessaloniki, Greece; 6 Laboratory of Microbiology, Hippokration General Hospital, 54642 Thessaloniki, Greece

**Keywords:** Candidemia, Epidemiology, Antifungal resistance, *Candida* species, Intensive care unit

## Abstract

**Background:**

Candidemia is a life-threatening invasive fungal infection, particularly in patients admitted to intensive care units (ICUs). Epidemiology of candidemia and antifungal resistance have been significantly affected in COVID-19 pandemic.

**Methods:**

We analysed candidemia cases in neonatal, paediatric, and adult ICUs at Hippokration General Hospital in Thessaloniki, Greece (site 1) and Kayseri City Training and Research Hospital in Kayseri, Türkiye (site 2) from January 2020 to December 2023. Epidemiology, species distribution, and antifungal susceptibility of cases were compared.

**Results:**

A total of 388 patients from site 1 and 379 patients from site 2 were included. A significant increase in the incidence of candidemia was observed in both hospitals during the COVID-19 pandemic. *Candida parapsilosis* was the most common species in all ICUs at Site 1, while *Candida albicans* was predominant at Site 2. *C. parapsilosis* became the most frequent species at Site 2 after 2021. *C. glabrata* was isolated more frequently in Site 1, whereas *C. tropicalis* was more frequently isolated in Site 2. A total of 14 *C. auris* strains were isolated, 13 of which were at the site 1. Over 90% of *C. albicans* strains were fluconazole-sensitive, but resistance was high in *C. parapsilosis* strains (47% at the site 1 and 40% at the site 2).

**Conclusion:**

*C. parapsilosis* and *C. albicans* remained the most common species, but their distributions varied between the two locations and over time. The high fluconazole resistance in *C. parapsilosis* causes a significant challenge for treatment. These findings highlight the necessity of continuous epidemiological surveillance to ensure appropriate antifungal treatment in different geographical regions.

## Introduction

Candidemia is a potentially life threatening invasive fungal infection caused by *Candida* species entering the bloodstream and is an important cause of morbidity and mortality in patients of all ages [1]. In recent years, the incidence of candidemia has been increasing in hospitalized patients, especially those in intensive care units [2]. This upward trend of incidence is associated with factors such as increase in the number of immunocompromised individuals, frequent use of broad-spectrum antibiotics, use of central venous catheters, prolonged hospitalisation of critically ill patients, surgical interventions and invasive medical procedures [3, 4]. In addition, there are reports that COVID-19 may have increased the incidence of candidemia [5, 6].

Although *Candida albicans* has historically been the most frequently isolated species, an increase in the prevalence of non-*albicans Candida* species has also been recently reported in many different settings and geographic areas [7, 8]. Differences in the antifungal resistance profiles among these species with variable prevalence in the world further complicate treatment management [9, 10].

In this study, we compare the epidemiological characteristics of candidemia, distribution of *Candida* spp. and antifungal susceptibilities in critically ill patients of two tertiary-care hospitals in different countries during and after COVID-19 pandemic.

## Materials and Methods

### Patients

This retrospective study was conducted in Hippokration General Hospital, Thessaloniki Greece (Site 1) and Kayseri City Training and Research Hospital, Kayseri, Türkiye (Site 2). Candidemia cases in Intensive Care Units (ICUs) of the two hospitals between January 2020 and December 2023 were identified and 4-year epidemiology, species distribution and antifungal susceptibility were determined. Patients of any age with *Candida* spp. detected in at least one blood culture per patient in neonatal, pediatric and adult ICUs were included in the study. Age and gender of the patients, type of ICU, isolated *Candida* spp. and susceptibility to antifungal drugs were pseudanymized and recorded in Excel database.

Growth of the same organism in a patient at least 14 days after a recorded episode or isolation of different *Candida* spp. any time after a previous episode was considered as a new episode. Duplicate isolates from the same episode were excluded according to the predefined episode criteria. No additional exclusion criteria were applied.

The ICU structures of the participating hospitals differed. Site 1 consisted of one adult ICU with a total capacity of 37 beds, one pediatric ICU with 8 beds, and two neonatal ICUs with a total capacity of 40 beds. Site 2 consisted of six adult ICUs with a total capacity of 68 beds, one pediatric ICU with 14 beds, and one neonatal ICU with 42 beds.

### *Candida* spp. isolation, species identification and antifungal susceptibility test

In site 1, blood culture bottles were incubated in BacT/ALERT 3D system blood culture incubator (bioMeriéux, Inc. Marcy l’Etoile, France) until a positive signal or up to five days. When yeast cells were observed on Gram stained slides prepared from blood culture bottles with a positive signal, subcultures were performed on Sabouraud dextrose agar (SDA) and incubated at 35–37 °C in aerobic medium for up to 5 days. During the time of the study, identification was performed by germ tube test and VITEK® 2 (bioMérieux, France). The susceptibility of the strains to caspofungin, micafungin, fluconazole, voriconazole, flucytocine and amphotericin B was determined using the VITEK® 2 *Candida* system. For determination of the minimum inhibitory antifungal drug concentrations of *Candida auris* specifically, Sensititre YeastOne assay (Thermo Fisher scientific, USA) was used.

In site 2, blood culture bottles were incubated in a BACTEC™ 9240 blood culture device (Becton Dickinson, Sparks, MD, USA) until a positive signal or up to five days. When yeast cells were observed on Gram stained slides prepared from blood culture bottles with a positive signal, they were passaged on Sabouraud dextrose agar (SDA) and incubated at 35–37 °C in aerobic medium for up to 5 days. Species identification was performed by germ tube test, *Candida* CHROMagar™ (Becton Dickinson, Sparks, MD, USA) and VITEK® 2, and morphological images were obtained for the isolates grown on Tween-80-corn-meal agar. Isolates were tested for susceptibility against fluconazole (FLC), amphotericin B (AMB), caspofungin (CAS), and voriconazole (VRC) by the E-test (bioMérieux, France) and microdilution methods.

The results were evaluated according to The European Committee on Antimicrobial Susceptibility Testing (EUCAST). Since clinical cut-off values for amphotericin B could not be determined, the results were interpreted according to epidemiological thresholds [11].

Different commercial systems were used for antifungal susceptibility testing in the two participating centers according to local laboratory protocols.

### Statistical analysis

Statistical analyses were performed using SPSS software version 22.0 (IBM Corp., Armonk, NY, USA). Categorical variables were compared using the chi-square or Fisher’s exact test, as appropriate. A p value < 0.05 was considered statistically significant.

## Results

A total of 388 patients (33 neonates, 32 children, 323 adults) from Site 1 and 379 patients (31 neonates, 46 children, 302 adults) from Site 2 developed at least one episode of candidemia during the study period. The median (min–max) ages in adult, pediatric and neonatal patients were 67.0 (17.0–92.0) years, 4.5 (1.0–15.0) years, 1.4 (0–7) months in Site 1 and 74.0 (17.0–99.0) years, 4.0 (1.0–17.0) years, 1.6 (0–9) months in Site 2. Sixty-one percent of the patients in site 1 and 55.4% in Site 2 were male (p = 0.11).

**Fig. 1:**
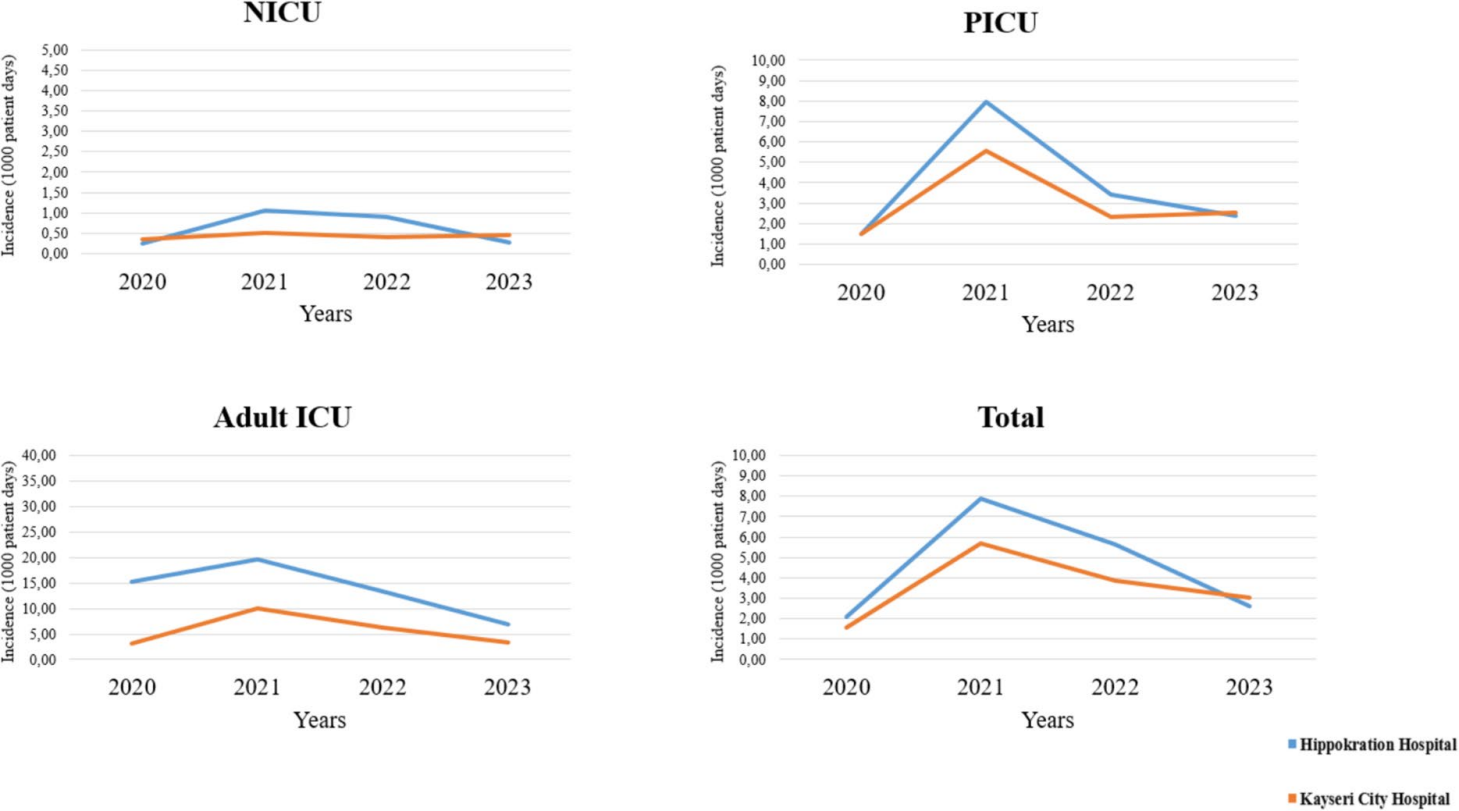
Incidence (per 1000 patients/days) of candidemia in different departments of the two hospitals. NICU: neonatal intensive care unit; PICU: pediatric intensive care unit

Overall 4-year incidence of candidemia was 4.59 episodes per 1000 patient-days in site 1 and 3.39 episodes per 1000 patient-days in site 2. Both sites had more than a triple increase of the annual incidence of candidemia in ICUs during 2021 (7.9 episodes per 1000 patient-days in site 1 and 5.7 episodes per 1000 patient-days in site 2 during 2021), which was followed by a decrease within the next two years. Of note, this pattern predominated mainly in adult and pediatric ICUs and not in neonatal ICUs (Fig. 1).

**Fig. 2:**
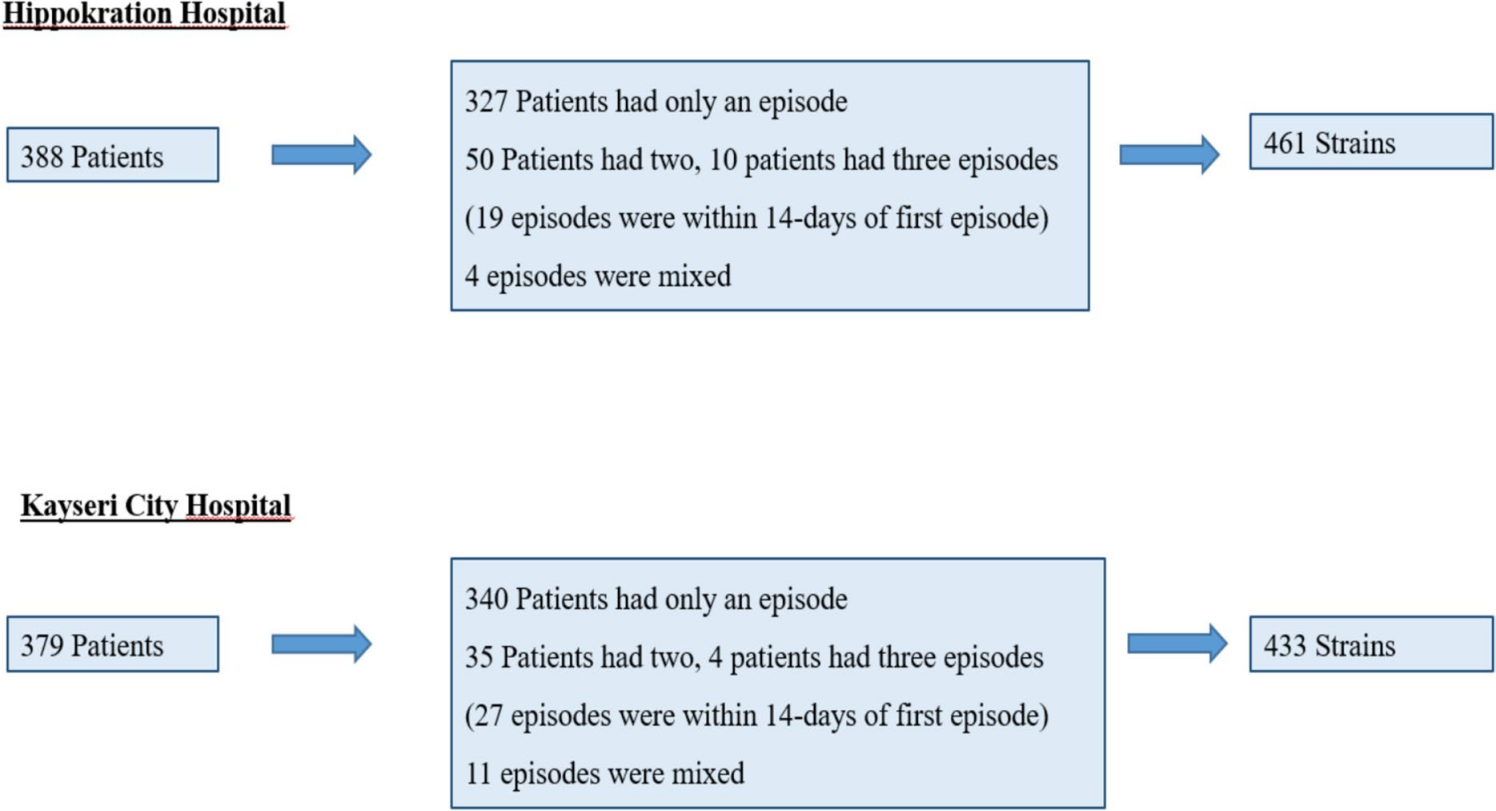
Patients infected and strains of *Candida* spp. isolated from these patients

In total, 461 strains were isolated in site 1 and 433 were isolated in site 2 (Fig. 2). In site 1, 327 patients had only one episode, 50 patients had two, 10 patients had three episodes and four episodes were mixed. In site 2, 340 patients had only one episode, 35 patients had two, four patients had three episodes and 11 episodes were mixed.

**Fig. 3:**
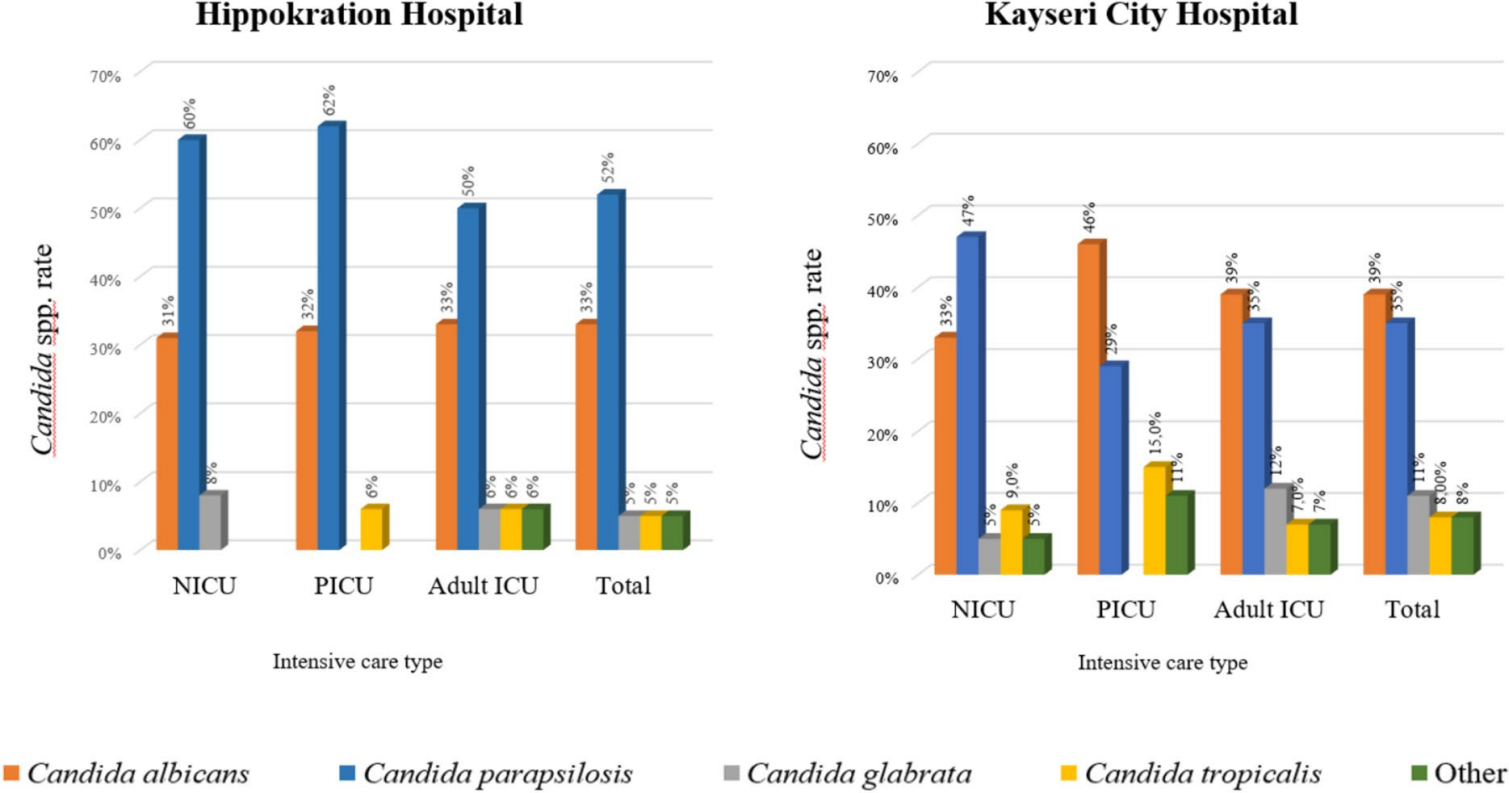
Distribution of *Candida* spp. isolates in the different departments of the two hospitals. NICU: neonatal intensive care unit; PICU: pediatric intensive care unit

**Fig. 4:**
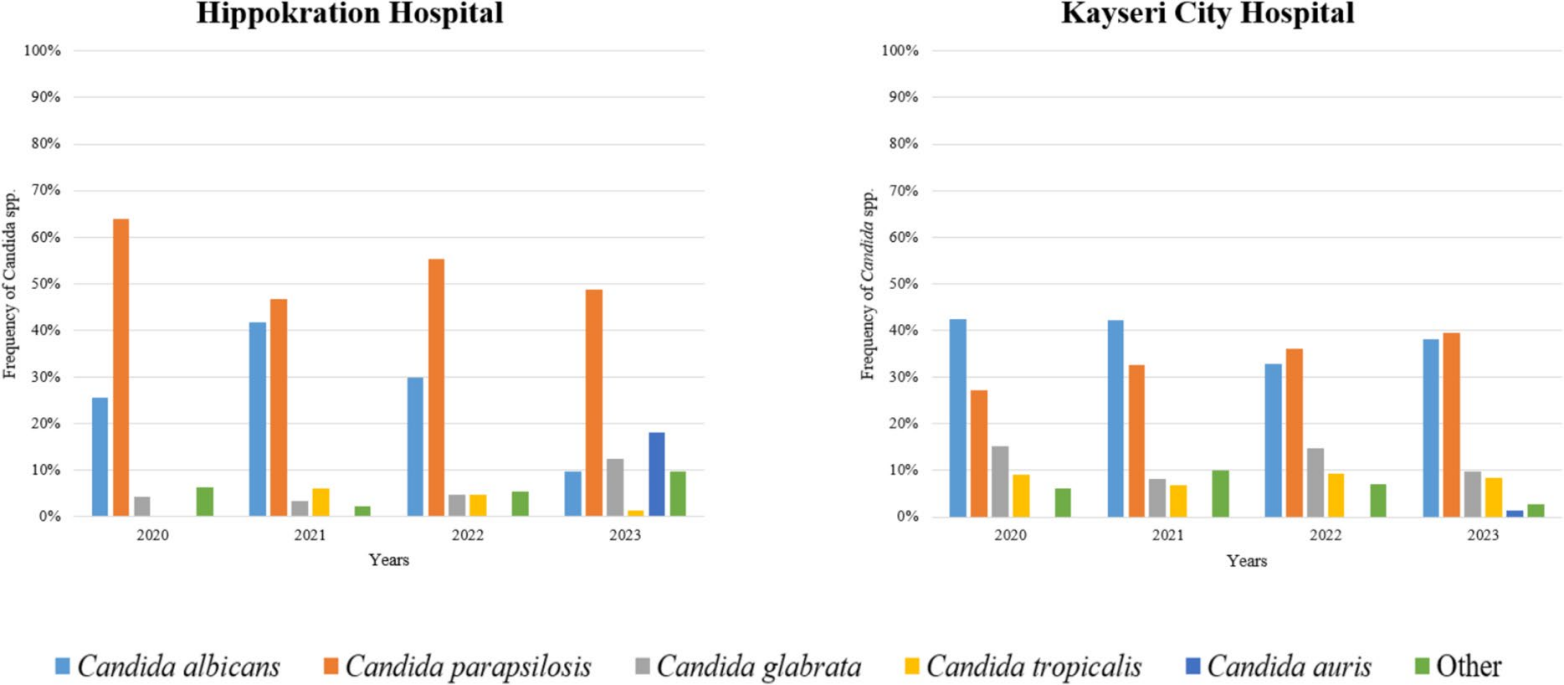
Prevalence of *Candida* spp. in the two hospitals by year of observation

*C. parapsilosis* was the most common *Candida* species in all types of ICUs in Site 1 and only in the NICU in Site 2. In pediatric and adult ICUs of Site 2, *C. albicans* was the most common species (Fig. 3). *C. parapsilosis* was the most prevalent isolate (> 45%) in all NICUs. *C. glabrata* (Nakaseomyces glabratus) was isolated more frequently in Site 2 than in Site1 (11% vs 5%), whereas *C. tropicalis* was slightly more frequent in Site 1 than in Site 2 (approximately 6% vs 5%). A total of 14 *C auris* (*Candidozyma auris*) strains were isolated, all but one in site 1 (Fig. 4).

**Fig. 5:**
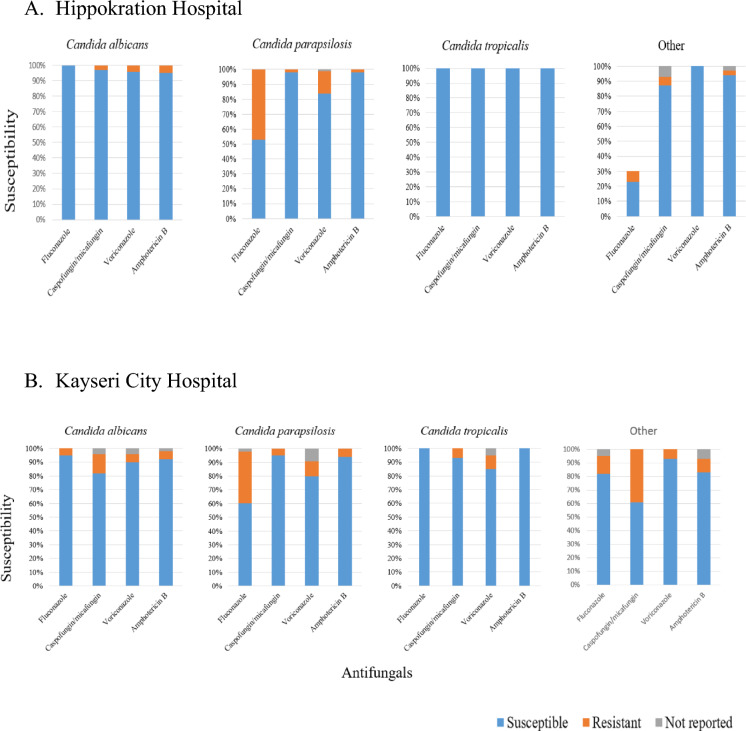
Reported susceptibility profiles to amphotericin B, fluconazole and micafungin for the most common *Candida* species. (**A**) Hippokration Hospital (**B**) Kayseri City Hospital

In both sites, more than 90% of *C. albicans* strains were susceptible to fluconazole, while almost half of *C. parapsilosis* were resistant. Antifungal susceptibility rates of most frequent *Candida* spp. are presented in Fig. 5. The median MIC values of the strains against antifungal drugs are given in Table 1.

**Table 1:** Minimum inhibitory concentrations of isolated *Candida* spp

*Candida* species	Fluconazole			Caspofungin			Voriconazole			Amph B		
	MIC_50_	MIC_90_	Range	MIC_50_	MIC_90_	Range	MIC_50_	MIC_90_	Range	MIC_50_	MIC_90_	Range
*Candida albicans* (n = 172)	2.0	4.0	0.064–8	0.5	1.0	0.012–2	0.25	0.75	0.01–2	0.25	0.5	0.002–3
*Candida parapsilosis* (n = 144)	2.0	8.0	0.12–256	1.0	2.0	0.002–6	0.25	1.0	0.01–3	0.38	1.5	0.002–4
*Candida glabrata* (n = 50)	24.0	64.0	0.32–256	0.5	1.0	0.125–3	0.75	2.0	0.013–32	0.75	1.5	0.06–2
*Candida tropicalis* (n = 35)	2.0	4.0	0.5–6	0.5	1.0	0.016–2	0.25	0.5	0.01–2	0.25	1.0	0.032–2
*Candida auris* (n = 13)	128.0	256.0	64.0–256.0	0.06	0.12	0.015–0.12	0.5	1.0	0.12–1.0	0.5	1.0	0.25–1.0
Other (n = 23)	2.0	8.0	0.25–48.0	0.5	2.0	0.15–6.0	0.25	0.5	0.016–2.0	0.25	0.75	0.016–2.0

## Discussion

In this study, the four-year epidemiology of candidemia in neonatal, pediatric and adult intensive care units of two tertiary care hospitals located in two different but neighboring countries was evaluated. We found that the incidence pattern of candidemia was similar over the years in the two sites. There were differences in the epidemiology of the most common *Candida* subtypes and their distribution in the intensive care units of the two institutions.

During the study period, the most striking point was the peak of candidemia incidence in both sites in 2021. There are previous studies reporting an increase in the incidence of candidemia during COVID-19. In a 19-year report from China published, a similar peak in incidence was observed in 2021 [12]. Araujo JM. et al., reported that in 2020, the incidence of candidemia per 1000 patient-days in Brazil increased by 38% [13]. Machado et al*.*, showed that the incidence of candidemia was higher in patients with COVID-19 than in controls, both as number per 1000 patient-days (3.22 vs 1.14) and as number per 1000 admissions (4.73 vs. 0.85) [5). In a prospective multicenter cohort study conducted in intensive care units in France, Reizine et al*.* reported an incidence of candidemia in COVID-19 patients higher than non-COVID-19 patients [14]. In general, this increase has been attributed to the increased need for intensive care and invasive procedures associated with COVID-19, as well as increased use of corticosteroids [15]. In this regard, our findings are in line with previously published observations reporting increased candidemia incidence during the COVID-19 pandemic period.

Of note, in 2021 during the peak of the pandemic the incidence of candidemia followed a similar trend both overall and in particular in the PICUs and adult Intensive Care Units. *C. parapsilosis* was the most common species of *Candida* in all intensive care units at Centre 1; by contrast, at Centre 2, it was the predominant species only in the neonatal intensive care unit. *C. tropicalis* was more frequently reported in site 2 than in site 1. In addition, *C. parapsilosis* was the most common species at both sites in 2022 and 2023. Our results regarding *C. parapsilosis* echo epidemiological trends from other countries as well in neonatal and pediatric settings [16, 17].

Differences in ICU organization and patient populations between the two participating hospitals may also have contributed to the observed differences in *Candida* spp. distribution. In particular, variation in the relative distribution and capacity of neonatal, pediatric, and adult ICUs may have influenced the predominance of *C. parapsilosis* and *C. albicans* between the two centers.

According to recent epidemiological reports about candidemia in the literature*,* different *Candida* spp. can be seen with varying frequencies among age groups. Although *C. albicans* is the most frequently isolated subspecies in children, *C. parapsilosis* may be more common in the neonatal group due to the increased use of central catheters, immature immune systems, prolonged hospital stays, parenteral nutrition, and frequent use of broad-spectrum antibiotics and steroids [18–20]. The most prominent subspecies in the changing epidemiology is *C. auris*. It is an emerging yeast species that is spreading rapidly worldwide and poses a serious public health threat because of its resistance to multiple antifungal drugs [21]. The increasing incidence of *C. auris* has brought this pathogen to the forefront in recent years. Cases and outbreaks have been reported in many countries, including Greece and Türkiye [22, 23]. During the study period, 13 *C. auris* isolates were detected at Site 1 [24], whereas only one isolate was identified at Site 2. This marked difference may reflect variations in local epidemiology and institutional outbreak dynamics, particularly considering the recent increase in *C. auris* outbreaks reported in tertiary-care hospitals in Greece during and after the COVID-19 pandemic [22–24]. Although antifungal resistance rates vary between species, regions and sites, resistance to fluconazole is increasing worldwide. Recently, concerns have been raised in the literature about the increase in fluconazole-resistant *C. parapsilosis* strains. In a reference laboratory in the USA, fluconazole resistance in 1,740 C. parapsilosis blood isolates analyzed was shown to increase from 8.2% in 2015 to 20.3% in 2024 [25]. Additionally, the multicenter CANDIMAD study conducted in Madrid showed that the fluconazole resistance rate in blood isolates increased from 2.6% in 2019 to 36.6% in 2022 [26]. In a meta-analysis of 71 studies focusing on fluconazole resistance, the resistance rate was 11.6% on average before 2016 and increased to 36.7% between 2016 and 2022 [27]. Fluconazole is an easily accessible, safe and inexpensive drug, very important especially in low and middle income countries, and the development of resistance leads to difficulties in empiric treatment and limits the armamentarium of antifungal drugs to more expensive or toxic agents. In our study, 47% of *C. parapsilosis* strains were resistant in Site 1 and 40% in Site 2 (Fig. 5).

The distribution of major *Candida* spp. can vary significantly across geographical regions and over time. While the incidence of candidemia has increased during the COVID-19 pandemic [5, 6, 28], in many publications, the most common subtype of both pre-pandemic and COVID-related candidiasis worldwide remains to be *C. albicans*. However, there has been a significant and serious increase in prevalence of non-*albicans Candida* species in many geographical regions [24, 29, 30]. Our study showed that *C. parapsilosis* was the most frequently observed species in Site 1 over the years. At Site 2, *C. albicans* was the most common species during the 2020–2021 period, while *C. parapsilosis* was the most frequently detected species during the 2022–2023 period. An important limitation of this study is the use of different commercial systems for blood culture processing, species identification, and antifungal susceptibility testing in the two participating centers. In addition, antifungal susceptibility testing results were not confirmed by reference broth microdilution methods according to CLSI or EUCAST standards. Therefore, discrepancies in MIC determination and resistance categorization may have occurred, particularly among isolates with borderline MIC values. Furthermore, because of the retrospective multicenter design, detailed demographic and clinical characteristics were not uniformly available for all patients. These limitations should be considered when interpreting inter-center comparisons and antifungal resistance rates.

## Conclusion

Candidiasis is a common infection, especially in intensive care units. The distribution of causative microorganisms and antifungal susceptibility rates may vary over time among clinics, sites and regions. Up-to-date epidemiological information on species epidemiology and resistance is important for the appropriate early treatment of patients.

## Data Availability

No datasets were generated or analysed during the current study.
